# Characteristics of hematopoietic stem cells of umbilical cord blood

**DOI:** 10.1007/s10616-014-9796-y

**Published:** 2014-11-06

**Authors:** Anna Hordyjewska, Łukasz Popiołek, Anna Horecka

**Affiliations:** 1 Department of Medical Chemistry, Medical Univeristy of Lublin, Lublin, Poland; 2 Department of Organic Chemistry, Medical Univeristy of Lublin, Chodźki 4A Street, 20-093 Lublin, Poland

**Keywords:** Stem cells, Cord blood, CD34+ cells, Cytokines, Cell cultures

## Abstract

Umbilical cord blood collected from the postpartum placenta and cord is a rich source of hematopoietic stem cells (HSCs) and is an alternative to bone marrow transplantation. In this review we wanted to describe the differences (in phenotype, cytokine production, quantity and quality of cells) between stem cells from umbilical cord blood, bone marrow and peripheral blood. HSCs present in cord blood are more primitive than their counterparts in bone marrow or peripheral blood, and have several advantages including high proliferation. With using proper cytokine combination, HSCs can be effectively developed into different cell lines. This process is used in medicine, especially in hematology.

## Hematopoiesis

**Fig. 1:**
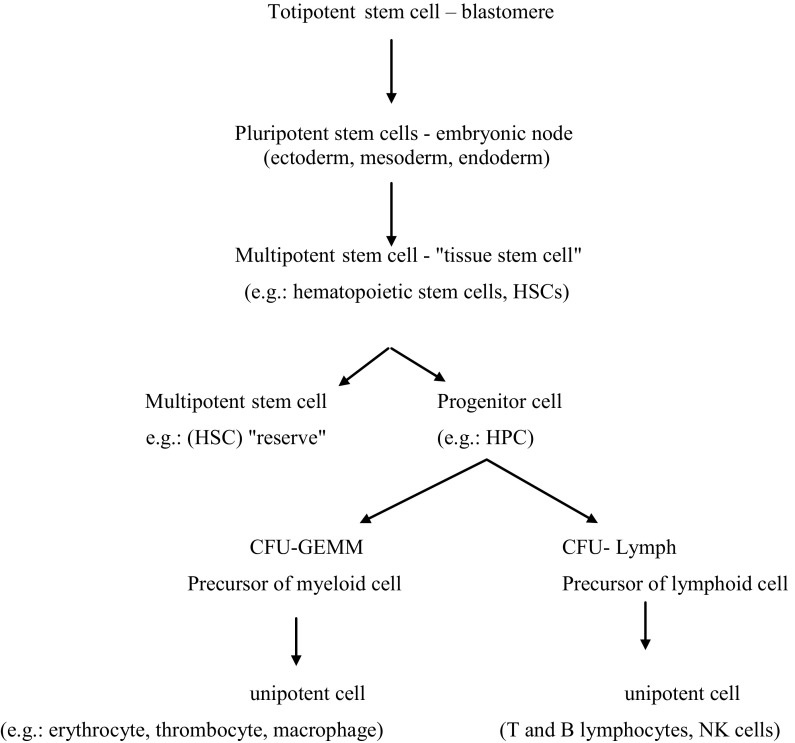
Formation of hematopoietic cell lines (Dąbrowski 1998a; Stojko and Witek 2005; Yao et al. 2004)

Blastomeres are the first stem cells in the development of the human body. These cells have the ability to develop into any cell type of the body, so-called totipotency. In a further development of the embryo—gastrulation stage, cells lose their properties of totipotency and begin the speciation process. When the embryo develops to the stage of gastrula two types of cells are formed: the throphoblast and embryonic node. Cells present in the embryonic node are called pluripotent cells—they have the ability to transform into all types of cells derived from the ecto-, meso- and endoderm. After moving to the uterus, these cells cannot differentiate into germ cells in the placenta and the surrounding tissue (Stec et al. 2003). In the further stages, pluripotent cells are transformed into “tissue stem cells”, so-called multipotent cells. These cells are subdivided into two progenies: one parent stem cell and one daughter cell, which has unipotent activity. The development process of stem cells is most readily observed in the hematopoietic system (Fig. 1) (Jósiak et al. 2004). All blood cell elements have their origin in a small population of hematopoietic stem cells, that have the ability to self-replicate, self-renew and to differentiate into specific cell lines (Yao et al. 2004).

Fetal hematopoiesis starts at about 2–3 weeks after fertilization and, initially, takes place in the yolk-sac. During fetal life, hematopoiesis gradually moves to the liver, and then after the development of the bones, at about 5–6 weeks takes place in the bone marrow (Czajka et al. 1999; Jędrasiak et al. 1999). A multipotential hematopoietic stem cell (HSC) during whole life divides asynchronously into two daughter cells—one HSC and one hematopoietic progenitor cell (HPC). HPC is the earliest progenitor cell, which, unlike the HSC, does not have the capacity to self-renew and is limited to one or more extra lines of differentiation (Kucia and Drukała 2002) “where there is no return,” and is removed during programmed cell death (Dąbrowski 1998a; Grskovic et al. 2004). HPC may give rise to myelocytic precursor colony forming unit of granulocyte, erythroid, macrophage and megakaryocyte (CFU-GEMM) or lymphoid precursor (CFU-Lymph). The targeted cells defined by some authors as colony forming cells (CFC) originate from CFU-GEMM or CFU-Lymph (Jędrasiak et al. 1999).

Colony forming unit of granulocyte, erythroid, macrophage and megakaryocyte gives rise to cells such as: colony forming unit of erythroid (CFU-E), colony forming unit of megakaryocytes (CFU-Meg), colony forming unit of granulocytes and macrophages (CFU-GM), colony forming unit of eosinocytes, colony forming unit of basophiles, colony forming unit of mastocyte, form which the erythrocytes, platelets, neutrophils, monocytes, macrophages, eosinophils, basophils, and the mast cells are generated, respectively. B cells, NK cells and precursors of thymocytes are formed from lymphoid precursor (Dąbrowski 1998a).

## The morphology of cord blood hematopoietic cells

The morphology of human HSC is similar in appearance to a small cell with a narrow hem cytoplasm, in which mitochondria and endoplasmic reticulum are poorly marked (Kopeć-Szlęzak and Podstawka 2001). It has the ability to intense proliferation and self-renewal and the ability to multi-line differentiation (Belvedere et al. 1999; Brunet de la Grange et al. 2002; Summers et al. 2001; Thierry et al. 1992). HSC is maintained in the G_0_ phase of the cell cycle, does not exhibit metabolic activity and has almost totally inhibited protein synthesis (Machaliński et al. 1998), thereby it is slightly stained with fluorescent dyes, such as Rhodamine 123, Hochest 33342, or Pyronin Y (Dravid and Rao 2002; Machaliński et al. 1997; Machaliński and Ratajczak 1997; Machaliński et al. 1998).

Activation of hematopoietic cells is combined with its output from G_0_ phase to G_1_ phase, which is characterized by increase of transcription and mRNA accumulation. Cells derived from long-term cultures have similar morphology: they are large and round, with large and round nucleus, have a small amount of cytoplasm, which is also characteristic for HPC progenitor cells (Tian et al. 2005).

## Surface markers’ expressing on cord blood hematopoietic cells

For several years, the surface marker—CD34 antigen, was only used to determine the hematopoietic cells. Nevertheless, most cells with CD34 antigen expression of bone marrow or umbilical cord blood, have other antigenic determinants. The immunophenotype of stem cells/progenitors can be assessed using:

- the cytometric analysis of the presence of CD34/CD38 proteoglycan
- analysis of the marker of mature line (HLA-DR)
- analysis of c-kit tyrosine kinase receptor and their respective labeling with the antibodies conjugated to fluorochromes (Stojko and Witek 2005).

As it is mentioned above, a characteristic feature of hematopoietic stem and progenitor cells is the presence of CD34 antigen. It is a transmembrane glycoprotein of approximately 104–120 kDa, which belongs to the adhesion molecules known as sialomucines. It is composed of a protein core of 40 kDa containing 6 to 9 *N*-binding sites of glycosylation and more than 9 *O*-binding sites of glycosylation (Tarach 1999). The cytoplasmic part of CD34 antigen has two sites for the phosphorylation of protein kinase C, and one site for tyrosine phosphorylation. Therefore its function is associated with the occurrence of transmembrane signalling (Tarach 1999). CD34 antigen is involved in the regulation of hematopoietic stem cell’s adhesion to the stroma (Gutierrez-Rodriguez et al. 2000; Kopeć-Szlęzak and Podstawka 2001). The increase in the expression of CD34 antigen, mainly precedes cell differentiation. Over 10 years ago, Gallacher et al. (2000) have isolated CD34− cells form mouse bone marrow, which possessed the ability to repopulate with 300 times higher efficiency than CD34+ cells. This so-called side population (SP) has also been found in pigs, monkeys, and in bone marrow and umbilical cord blood of people. After 3–5 weeks in culture SP-cells become mostly CD34+ cells. Therefore there is a hypothesis of the presence of a “stem cell cycle”, which implies the possibility of a reversible phase transition form CD34− to CD34+ (Gallacher et al. 2000; Tarach 1999).

DNA testing has shown that cells having antigen CD34+, being in S phase, or G_2_/M cell cycle, show increased expression of the CD38+ antigen, which reflects their activation (Belvedere et al. 1999; Kopeć-Szlęzak and Podstawka 2001; Qian-Lin et al. 1995). Cells expressing the CD34+CD38− antigens are inhibited in the G_0_ phase. CD38+ antigen determines more diverse cells than those without this marker: CD34+CD38− are more primitive than CD34+CD38+ (Summers et al. 2001; Tian et al. 2005). Another antigen used to determine the degree of maturity is CD90 antigen, also known as Thy-1. It occurs on primitive cells or early progenitors, and its lack is observed on late progenitors. Studies have shown that the number of CD34+CD90+ cells correlates positively with the number of CD34+CD38− cells.

Co-expression of CD117 and CD135 antigens (with tyrosine kinase receptor properties) is also used for the determination of the maturity of early CD34+ cells. More than 60 % of the population of CD34+ cells shows co-expression of CD117 antigen, which is also known as the c-kit receptor for the stem cell factor (SCF) (Dąbrowski 1998a; Ruzicka et al. 2004). Primitive CD34+ cells generally have low expression of this antigen, in contrast to the progenitor cells, where its higher expression is observed. 90 % of CD34+ cord blood cells show expression of CD135 antigen with properties of tyrosine kinase, and 25 % of CD34+ cells show the CD95 antigen expression, which probably acts as a regulator during early hematopoiesis (Kopeć-Szlęzak and Podstawka 2001). Antigen CD71 is the transferrin receptor, which is situated on the surface of hematopoietic cells. The primary lack of this antigen on primitive cells and its later appearance on them, suggests that these cells will be differentiated into the line of CFU- E (the characteristic antigen pattern for this line is CD34+CD45−CD71+) (Fasouliotis and Schenker 2000, Kopeć-Szlęzak and Podstawka 2001). At the very early stage of differentiation, co-expression of CD45RO and CD45RB antigens exists, without expression of the CD45RA antigen. This antigen is characteristic of late progenitors, and suggests that CFU-GM cell line will develop (Fasouliotis and Schenker 2000; Kopeć-Szlęzak and Podstawka 2001) (immunophenotype CD34+CD45RA+CD71−).

On the CD34+ cells there is also found the AC 133 antigen. It is a glycosylated protein with molecular weight of 120 kDa. It does not show homology to any of the previously described antigens present on hematopoietic cells—the structure is unique among other cell surface antigens. It has an extracellular N-terminus, which is a 5-transmembrane domain and the cytoplasmic C-end. This antigen appears to play role of a growth factor receptor. The presence of the five C-terminal tyrosine on it, indicates that this protein is phosphorylated in response to attachment of the ligand (Miraglia et al. 1997). AC133 expression is shown mainly by CD34+ Lin- cells, but there is also evidences of the presence of this antigen in the population of CD34-Lin- cells (Gallacher et al. 2000; Grskovic et al. 2004).

To assess the proliferative potential of already phenotypically identified hematopoietic cells—clonogenic assays (e.g.: CFU-GM, CFU-M, BFU-E) and culture assays (LTC-IC, Long Term Culture Initiating Cell) are needed (37, 44). Grskovic et al. suggest that colony forming cells (CFC) in ex vivo cultures are derived from CD34+ cells with low co-expression of c-kit (CD117). Additionally, the presence of CD135 antigen has influence on the proliferative-differential activity of these cells. Smogorzewska et al. have noticed that CD34+CD38− cells were able to form colonies (CFU) after 60 days of co-culture in the presence of bone marrow stroma, unlike to CD34+CD38+ cells, which were not able to form CFU in culture beyond 40 days.

## Differences between cord blood, peripheral blood and bone marrow

### Differences in cell’s content and composition

Cord blood cells differ from their counterparts in the peripheral blood or bone marrow, both in composition, number, and properties (Mariańska 1997; Qian-Lin et al. 1995, Thierry et al. 1992; Zhou et al. 2001). Cells in cord blood (with a CD34+CD38− phenotype) which are found in the G_0_ phase have greater proliferative response to cytokines and are less dependent on stromal cells than the corresponding cells in the bone marrow or peripheral blood (Kopeć-Szlęzak and Podstawka 2001; Smogorzewska et al. 1997; Zhou et al. 2001). Studies of Gao et al. indicate that in the cord blood there are hematopoietic precursors of stroma (Gao et al. 2006). In the umbilical cord blood, HPP-CFC cells (high proliferative potential colony forming cell–cells with high proliferative potential, capable of forming colonies) are also present in an amount exceeding eight times their content in bone marrow (Czajka et al. 1999; Mariańska 1997, 2000; Stojko and Witek 2005). Analysis of CFCs present in the human umbilical cord blood shows that in 1 ml of blood there is about 8,000 of BFU-E (i.e. 3 times more than in the bone marrow or peripheral blood) between 13,000 and 24,000 of CFU-GM (i.e. 15 times higher concentration than in the bone marrow or peripheral blood) and between 1,000 and 10,000 of CFU-GEMM (Brunet de la Grange et al. 2002; Fasouliotis and Schenker 2000). In addition, cord blood contains a higher proportion of more primitive hematopoietic cells than bone marrow (Smogorzewska et al. 1997). The value of cord blood cells expressing on their surface CD34 antigen is approximately 0.02–1.43 %. This value is closed to the percentage of CD34+ cells found in the bone marrow of adult (0.5–5 %) rather than in the peripheral blood (<0.01 %) (Stolarek and Myśliwski 2005). The number of CD34 +HLA-DR—cells, and CD34+CD38− cells in the cord blood is higher than in the bone marrow (4 % in the umbilical cord blood and 1 % in bone marrow) (Stojko and Witek 2005).

On the CD34+ cells of umbilical cord blood, there is higher expression of CD44 proteoglycan and other adhesion molecules of integrins group such as CD49d or CD49f, but lower expression of CD11 and CD18 than in the same cells of the bone marrow or peripheral blood (Kopeć-Szlęzak and Podstawka 2001).

Cord blood cells are also characterized by long telomere DNA in comparison to their analogues from peripheral blood or bone marrow (Dąbrowski 1998a; Fasouliotis and Schenker 2000; Zhou et al. 2001). Therefore cells form cord blood are capable for hematopoiesis for a longer time—they generate more divisions and generate a larger number of progeny (daughter cells).

Cord blood cells, which belong to the immune system, also have many differences as compared with the corresponding cells in the bone marrow or peripheral blood (Jędrasiak et al. 1999; Zeman et al. 1996). The immunoreactivity of effector cells, such as lymphocytes and monocytes, and the total number of B lymphocytes are relatively close; however, the number of sub-populations of B cells is different. Half of the population are immature cells with the phenotype CD19+ CD5+. The total number of CD4+ and CD8+ cells is smaller, but the ratio of CD4+/CD8+ is greater than in peripheral blood (Zeman et al. 1996). Umbilical cord blood contains a characteristic T cell population (with phenotype CD3−/CD8−), which is the precursor of T cell line (Dąbrowski 1998a). A large number of studies indicate a lower percentage of NK cells in cord blood and consequently a lower cytotoxic activity as compared with the results obtained in adults (Zeman et al. 1996). Low level of natural cytotoxicity of umbilical cord blood cells is explained by “naive” feature of immune cells of umbilical cord blood and probably is a characteristic value of the perinatal and neonatal periods, which do not depend on exogenous factors (Jędrasiak et al. 1999; Kamiński et al. 1996; Pietruszczuk et al. 1998). CD34+ cells of cord blood are also more resistant to Pyronin Y staining as compared with the same fraction of bone marrow. It is explained by the fact, that the CD34+ cells in cord blood, under childbirth stress condition, are constantly stimulated by many cytokines, and therefore are less sensitive to the possible toxic environmental substances (Machaliński et al. 1998).

The presence, in umbilical cord blood, of mesenchymal stem cells that are precursors of certain types of cells (e.g.: bone, cartilage, fat or muscle) is no longer a controversial issue (Stolarek and Myśliwski 2005). Chang et al. showed that except for HSC, umbilical cord blood, similarly to bone marrow, contains mesenchymal stem cell (MPC—mesenchymal stem cell/mesenchymal cell progenitor) (Chang et al. 2006). Lee et al. have found that cord blood contains more primitive multipotential population MPC, which are able to differentiate into cells derived from the three germ layers (Lee et al. 2005). Chang et al. have noticed in cord blood two morphologically distinct phenotypes of MPC: the first described as lobar flattened fibroblast clones (F-MPC, about 93 %), the second as a spindle—shaped fibroblast clones (SS- MPC). In addition, researchers analyzed the surface markers of these two phenotypes of MPC. Both types were negative for antigen CD34, CD26, CD31, CD45 and HLA-DR. They showed, in turn, the expression of cell surface antigens typical for mesenchymal progenitors, such as: SH2, SH3 and SH4, adherent molecules of CD29, CD44 and HLA antigens—A, B, C. The only difference between these two types of MPC is the CD90 antigen expression: spindle shape-MPC exhibited high expression of the antigen, while flattened-MPC showed no CD90 expression (Chang et al. 2006).

### Differences in cytokine’s composition and production

Hematopoietic microenvironment is the place of origin of cell’s proliferation, cell’s differentiation and cell’s development. Fibroblasts, macrophages, osteoblasts, endothelial cells, and T cells (Th1 and Th2), which are present in this microenvironment, produce growth factors. The effect of these growth factors depends on the target cell, on the cytokine concentration and on the presence of other cytokines (Brunet de la Grange et al. 2002; Czajka et al. 1999; Majka et al. 2001; Verhasselt et al. 1999; Yao et al. 2004; Zhou et al. 2001).

In vitro studies revealed that CD34+ cord blood cells have higher affinity to stromal cell derived factor (SDF-1) than their counterparts in the bone marrow or peripheral blood (Kopeć-Szlęzak and Podstawka 2001; Ławicki et al. 2001). CD34+ cells of cord blood, in comparison with them, strongly react also on Interleukin 3 (IL-3), IL-6 and SCF, which brings about a considerably greater number of formed colonies (Cicuttini et al. 1993; Dąbrowski 1998b; Wagner et al. 1992).

Bogunia-Kubik has implicated that cord blood cells produce less cytokines in comparison with corresponding peripheral blood cells. The imbalance affects both the quantity of synthesized protein, its bioactivity and the number of cells producing cytokines. T cells, NK cells and macrophages of cord blood, produce small amounts of G-CSF, GM-CSF, IL-3, M-CSF, TGF-β, IL-4, IL-2, IFN-γ (Dąbrowski 1998b, Gao et al. 2006; Ławicki et al. 2001).

Limited cytokine synthesis by the cells of umbilical cord blood can be the result of:

- “naive” antigen feature (Bogunia-Kubik 2001)
- may be associated with decreased expression of mRNA, which encode a gene for cytokine, thereupon with less accumulation of cytokine’s mRNA and its volatility (Bogunia-Kubik 2001)
- reducing the production of cytokines, which may also be due to differences in signal transduction through the umbilical cord blood cells and peripheral blood and/or immaturity of the signal transduction mechanism in umbilical cord blood cells. According to some researchers the low response of umbilical cord blood cells is associated with a defect in the transmission of stimulatory signals through CD3 or with a defect in the TCR’s signal transduction. In contrast, other studies have shown, that decreased in vivo synthesis of cytokines by “naive” T cells (phenotype CD45RA+) from umbilical cord blood is not the result of a defect within the cells but rather high threshold of activation required by these cells (Bogunia-Kubik 2001)
- reduced production of cytokines, which may also be associated with suppressor properties of serum (Bogunia-Kubik 2001).

## The role of certain cytokines in the culture of umbilical cord blood cells

Each cell must dispose of the basic energy needs to differentiate into the appropriate type. Under physiological conditions, such situation is possible thanks to the interaction between cells responsible for maintaining of the homeostasis. In vitro conditions include appropriate methods of cell’s culture and the need to use certain media and grow factors. Since the beginning of the use of umbilical cord blood as source of hematopoietic stem cells, the development of techniques for obtaining cord blood cells and of culture methods have been reported. With using the appropriate “cocktail” of cytokines and specific media, it is possible to grow the cultures of human erythroblasts, megakaryoblasts and precursors of granulocyte–macrophage line (Lawicki et al. 2001; Stolarek and Myśliwski 2005).

An important issue is not only the type of factors, but also their concentration and sequence of administration. Stec et al. have found that providing (during the first 3 days) so-called “early cytokines” (SCF, IL-3, Flt3, Tpo) and a medium containing 4 % bovine serum for the HSC, and next the transfer these cells to medium containing 20 % bovine serum and growth factors such as: M-CSF, Flt3, IL-3, SCF cause the expansion of CD 14cells (Stec et al. 2003) (Table 1).

**Table 1:** Antigens expressed on HSC (Belvedere et al. 1999; Gutierrez-Rodriguez et al. 2000; Kopeć-Szlęzak and Podstawka 2001; Qian-Lin et al. 1995; Summers et al. 2001; Tarach 1999; Tian et al. 2005)

Surface marker	Occurrence
CD34	Stem cells, progenitor cells
CD38	Stem cells, early progenitor cells
CD90	Primitive cells, early progenitors
CD117	Progenitor cells
CD71	Precursor cells of CFU-E
CD45RA	Late progenitors of CFU-GM
CD135	Stem cells, progenitor cells
AC133	Stem cells

Growth factors, such as SCF, Flt3, IL-11, IL-3, IL-6, GM-CSF are responsible for the proliferation of the cells, while others, such as: M-CSF, G-CSF, Epo and Tpo are responsible for differentiation and maturation of cells. SCF, IL-3 and IL-6 are cytokines, which act in the G_0_/G_1_ phase of the cell cycle, and by working together induce mitosis (Grskovic et al. 2004) (Table 2).

**Table 2:** Differences between stem cells from different origins (Brunet de la Grange et al. 2002; Czajka et al. 1999; Fasouliotis and Schenker 2000; Kopeć-Szlęzak and Podstawka 2001; Mariańska 1997, 2000; Stojko and Witek 2005; Stolarek and Myśliwski 2005; Zeman et al. 1996)

Feature	Umbilical cord blood	Bone marrow or peripheral blood
Content of HPP-CFC cells	Higher	Lower
BFU-E content	8,000/ml	2,500/ml
CFU-GM	13,000–24,000/ml	870–1,600/ml
Expression of CD34+	0.02–1.43 %	0.5–5 % BM <0.01 % PB
Expression of CD34 + CD38-	4 %	1 %
Content of NK cells	Lower	Higher
Content of Th and Tc	Lower	Higher

### SCF

A stem cell factor (SCF)—is produced by bone marrow stromal cells, T lymphocytes, hepatocytes and fibroblasts. It reacts with the c-kit receptor, which is built of the extracellular SCF binding site, the transmembrane hydrophobic part and intracellular part with the tyrosine kinase activity. This receptor is present on the surface of bone marrow precursor cells and certain leukemic lines, in a number from 2,500 to 30,000 per cell (Dąbrowski 1998b) (Table 3).

**Table 3:** Ex vivo expansion of CD34+ CD38− cord blood cells due to implemented cytokines (Czajka et al. 1999; Dravid and Rao 2002; Jinquan et al. 2001; Kucia and Drukała 2002; Majka et al. 2001; Stec et al. 2003; Tian et al. 2005; Verhasselt et al. 1999)

Cells (phenotype)	Cytokines sets	Expansion of LTC-IC
CD34+CD38−	SCF, Flt3, IL-3, IL-6, IL-11, LIF,	HSC division
CD34+CD38−	SCF, Flt3, IL-3, GM-CSF, IL-6, IL-10	Progenitor cells—myelocytic line (CFU-GEMM)
CD34+CD38−	SCF, IL-4, IL-12, IL-15	Progenitor cells—lymphoid line (CFU-Lymph)
CD34+CD38−	SCF, SDF-1, Flt3, IL-13, IL-7, IL-10, IL-5	pro-, pre- B lymphocytes
CD34+CD38−	IL-3, IL-6, IL-2, IL-9, IL-1, IL-7	Thymocytes
CD34+CD38−	SCF, IL-3, IL-11, Tpo	Megakaryocytes (CFU-Meg)
CD34+CD38−	IL-3, IL-6, IL-11, IL-9, Epo, LIF	Erythrocytes (CFU-E)
CD34+CD38−	IL-3, IL-10, GM-SCF, M -SCF, IL-9	Macrophages (CFU-M)
CD34+CD38−	IL-3, IL-1, GM-SCF, G-CSF, IL-5	Granulocytes (CFU-G)
CD34+CD38−	SCF, IL-1, IL-3, GM-CSF, G-SCF, M-SCF	Granulocytes and macrophages (CFU-GM)
CD34+CD38−	IL-3, IL-4, IL-9, IL-10	Mastocytes (CFU-Mast)

Hematopoietic stem cell is the primary target for SCF, however, this factor stimulates the differentiation of HSCs only in a small percentage of cases. It is a good co-stimulator of growth and acts synergistically with other factors. SCF has influence on poorly differentiated stem cells, especially on hematopoietic cells, which are in early and intermediate stage of growth. In vitro, SCF works only when other growth factors are present in the medium (e.g.: IL-3, GM-CSF, G-CSF, Epo). For instance, it was observed that SCF interacts with EPO and stimulates therefore the erythroblasts colony formation, with GM-CSF or IL-3—it causes the formation of mixed colonies of early progenitor cells, with G-CSF—it stimulates neutrophiles colony formation (Dąbrowski 1998b), and in conjunction with IL-7—it stimulates the early stages of B cell differentiation (Dąbrowski 1998b; Ruzicka et al. 2004) (Table 4).

**Table 4:** Role of cytokines (Chen and Wang 2014; Dąbrowski 1998b; Jędrzejczak 1997; Jinquan et al. 2001; Li et al. 2010; Lawicki et al. 2001; Ruzicka et al. 2004)

Cytokine	Receptor	Origin of produce	Role
SCF	C-kit receptor	Bone marrow stromal cells, T lymphocytes, hepatocytes and fibroblasts	Co-stimulator of growth, stem cell division
IL-3	IL3-R or CD123	Activated T cells, NK cells, mast cells	Stimulates cell proliferation and differentiation of granulocyte-macrophages, erythrocytes, megakaryocytes, eosinophils and mast cell progenitors
IL-6	L-6R and gp130-R	Fibroblasts, activated T cells, activated monocytes, macrophages and endothelial cells	Increases the expression of most of cell surface antigens, which are characteristic for myeloid lines
GM-CSF	GM-CSF receptor	Activated T cells, endothelial cells, fibroblasts and mast cells	Stimulates the formation of granulocytes and monocytes colony
IL-9	IL-9R	Activated Th cells (Th2, Th9, Th17), mast cells	Induces the proliferation of various lymphoid and hematopoietic progenitors

### IL-3

Interleukin 3 is known as multipotential colony stimulating factor (multi-CSF), or mast cell growth factor (MC-GF). It is a glycoprotein with a molecular weight of 14–30 kDa. It is produced by activated T cells, NK cells, mast cells and some leukemia cell lines. IL-3 exerts its effect through the current cell receptor for interleukin 3 (IL3-R or CD123). IL-3 receptor is composed of two chains α and β, and shows a strong similarity to the GM-CSF receptor. IL-3 stimulates cell proliferation and differentiation of granulocyte–macrophages, erythrocytes, megakaryocytes, eosinophils and mast’s cell progenitors. The total number of CD123 on the surface of the progenitors mentioned above is about 200–13,000 per cell and decreases with their maturation, which indicates that IL-3 is a more proliferative cytokine than cytokines, which stimulate the functions of mature cells (Jędrzejczak 1997).

IL-3 acts synergistically with other growth factors, mainly with GM-CSF, G-CSF, IL-6 and IL-11. This is confirmed by in vitro studies, in which the addition of IL-3 with GM-CSF to the cell culture results in a larger number of granulocyte–macrophage colony than with sole GM-CSF (Lawicki et al. 2001). In combination with other cytokines, which act in the early stages of hematopoiesis, such as SCF, IL-1 or IL-6, it supports the formation of colonies and their expansion in culture (Ruzicka et al. 2004).

### IL-6

Interleukin 6 is a so-called inflammatory cytokine. It is produced by fibroblasts, activated T cells, activated monocytes, macrophages and endothelial cells. IL-6 increases the expression of most of cell surface antigens, which are characteristic for myeloid lines. IL-6 is a potent co-factor for the survival and proliferation of primitive progenitors and it acts on the cell by the interaction of two receptors: IL-6R and gp130-R. The stimulation with SCF, IL-6 and Epo induces rapid proliferation of selective stimulation of erythropoiesis. It has been shown that the addition of IL-6 to the medium without serum supplementation in a short-term culture had beneficial effects on HSC expansion of umbilical cord blood and also prevented the negative effect caused by IL-3 in the long-term culture (Ruzicka et al. 2004).

### GM-CSF

Granulocyte-macrophage colony stimulating factor (GM-CSF) is a glycoprotein with a mass of 14–35 kDa. It is produced mainly by activated T cells, endothelial cells, fibroblasts and mast cells. GM-CSF receptor belongs to the hematopoiethines family receptors without internal tyrosine kinase activity properties (Jędrzejczak 1997; Jinquan et al. 2001).

Large quantity of this receptor is found on the cells of the granulocyte line, but this amount changes during maturation. In vitro, GM-CSF stimulates the formation of granulocytes and monocytes colony. Recent studies have shown that GM-CSF induces on CD34+ cells the expression of CXCR3 (it is a receptor present on activated T cells and memory cells), and therefore has influence on the differentiation of CD34+ cells into appropriate type of precursor and their later maturation (Jinquan et al. 2001). In vivo, GM-CSF reduces the time of transition from resting phase to meiosis and S-phase duration, and affects lymphoid progenitor cells causing their proliferation and maturation (Lawicki et al. 2001; Tian et al. 2005).

### IL-9

IL-9 was first identified as a mouse T cell growth factor but with time it has also been demonstrated to modulate B cell maturation, IgE production and promote proliferation and differentiation of mast cells and hematopoietic progenitors. IL-9 is proposed to be a candidate gene for asthma (Chen and Wang 2014; Townsend et al. 2000) and with IL-3 it acts synergistically for maximal proliferation of mast cells. Therefore, IL-9 is described as a T cell derived cytokine with pleiotropic activities on various cell types. The action of IL-9 on thymocytes in vitro is interesting in view of the development of thymic lymphomas. In IL-9 transgenic mice it has been observed that IL-9 is a major anti-apoptotic factor for thymic lymphomas (Damera et al. 2006; De Smedt et al. 2000).

The functions of IL-9 are mediated by the IL-9 receptor (IL-9R), which is a member of the superfamily of hematopoietic receptors. The human IL-9R gene contains 11 exons and encodes a 522 amino acid protein. IL-9R is expressed in T-cell lines and effector T cells, but not in naive T cells. Among the Th cell subsets, IL-9R exhibits its highest expression in Th2 and Th17 cells (Chen and Wang 2014; Li et al. 2010).
